# Clinical Cases in Mercy Hospital

**Published:** 1874-11-01

**Authors:** Sam’l J. Jones

**Affiliations:** Professor of Ophthalmology and Otology in Chicago Medical College


### CLINICAL CASES IN MERCY HOSPITAL.

SERVICE OF SAM'L J. JONES, M. D., PROFESSOR OF OPHTHALMOLOGY AND OTOLOGY IN CHICAGO MEDICAL COLLEGE.

*Reported by J. R. Kewley.*

GENTLEMEN: This lady, a number of months ago, was seized with pain in the eye, soon followed by a reddened appearance over the region of the lachrymal sac. This inflammation of the lachrymal sac is sometimes mistaken for erysipelas, but unlike that disease the redness does not tend to spread; neither does it disappear upon pressure. The inflammation commences in the sac and extends more or less to surrounding parts. After pus is formed it frequently works its way through the tissues and discharges upon the external surface, thus forming a fistula. Several months ago such a fistula existed in this case, but, as you see, it is now entirely closed. You notice there is not any great accumulation of tears in the eye; this results from the lachrymal ducts having become impervious from the inflammation. To-day we will give exit to the pus by puncturing the swollen part, and direct the patient to apply warm water dressings to hasten its evacuation. When the pus is discharged we will slit up the canaliculus and probe the nasal duct, so that the secretions may escape into the nose as fast as they are formed.

### INFLAMMATION OF THE MEMBRANA TYMPANI AND MEATUS AUDITORIUS EXTERNUS.

This difficulty has existed for twelve months, resulting in impaired hearing, pain and fullness of the parts, with redness, and considerable excoriation. The mother does not give a clear description of its origin; but I think from the appearances it has been caused by eczema of the auricle and meatus. We will direct the mother to cleanse the parts twice daily with tepid water, by means of a syringe, using at least a pint of water each time.

SLIGHT OPACITY OF CORNEA.

Two years ago this young lady took cold, she says, in the eyes. Inflammation ensued, and as a result you see this slight cloudiness of both cornea. In these cases we generally use a crayon of sulphate of copper, in conjunction with an astringent collyrium. In this case we will direct a solution of two or three grains of sulphate of zinc, to the ounce of water, to be dropped into the eyes once daily.

CATARRHAL INFLAMMATION OF TYMPANUM.

This inflammation is the result of an ordinary cold, it having extended up through the eustachian tubes from the fauces. We will inflate the tympanic cavity, by means of the eustachian catheter, at the same time using the otoscope, in order not only to ascertain whether air enters the tympanum or not, but also to determine the condition of the mucous membrane lining the tympanic cavity. We will also force iodized air into the tympanum, thus obtaining all the benefit of a local application of iodine.

CHRONIC CONJUNCTIVITIS.

You will notice in this case the congested condition of the blood-vessels in the conjunctiva. Notice also the irregular course the vessels take, and the slight opacity of the cornea. This opacity results from a previous ulceration of the part. We will use the crayon of sulphate of copper.

OPHTHALMI TARSI.

There is present in this case, as you see, an inflammation of the edges of the eyelids, with more or less loss of the eyelashes. The great trouble in these cases is the adhesion of the lids, which is especially apt to occur during sleep. To prevent this adhesion taking place almost any oily substance is applied. I usually use a dilute citrine ointment, one part of this to five parts of rose ointment being my usual prescription, directing a little to be applied to the lids each night. In conjunction with this you may use a collyrium of sodæ bi-borate or zinc of sulphate.

TRAUMATIC CATARACT.

This old gentleman was struck in the eye with a large piece of metal. It was so large that it could not enter the eye, yet as a result we have here, as you notice, a cataract, the iris being in many places firmly adherent to the crystalline lens. A hernia of the iris also exists, pressing into the substance of the cornea. We will limit as much as possible the inflammation by applications of cold water, and keep the pupil dilated with a collyrium of sulphate of atropia until absorption has been completed.

---

An interesting case of chronic aortitis simulating angina-pectoris and producing neither œdema nor difficulty of breathing, has been recently reported to the Anatomical Society of Paris. The autopsy revealed excessive contraction of the orifices of the cervuary arteries, thickening of the kidneys and heart, and extensive atheromatous and calcareous deposit in the aorta, while it was noted as especially remarkable that in connection with these severe lesions of the aorta, the other arteries were found to be entirely intact.

